# Trying to clear the air: e-cigarette use and periodontal disease

**DOI:** 10.1038/s41415-025-8919-5

**Published:** 2025-11-28

**Authors:** Gabby Robson, Xii Cin Lim, Iman Chaudhari, Joshua Hurley, Saba Khalil, Veronica Amin, Luigi Nibali

**Affiliations:** 858263448176461236247 Undergraduate Student, Faculty of Dentistry, Oral and Craniofacial Sciences, King´s College London, London, United Kingdom; 323515363378144526991 Academic Foundation Dentist, University of Bristol, United Kingdom; 788345764346756852519 Foundation Dentist, London, United Kingdom; 911524735455058935676 Professor of Periodontology/Honorary Consultant, Periodontology Unit, Centre for Host Microbiome Interactions, Faculty of Dentistry, Oral and Craniofacial Sciences, King´s College London, London, United Kingdom

## Abstract

**Introduction** With record rates of e-cigarette use in the United Kingdom, it is becoming more important that dental professionals understand the consequences of e-cigarette use on oral health.

**Methods** This narrative review considers the current bank of literature regarding e-cigarette use and periodontal health and disease.

**Results** Some studies have found that e-cigarette users had worsened periodontal health and poorer response to periodontal treatment compared to non-smokers. However, there is mixed evidence on this topic and the current evidence base remains limited, with few high-quality studies available. Similarly, although current research suggests that e-cigarette use is safer for the periodontium than conventional smoking, further long-term, large-cohort studies will be required to improve the evidence base.

**Conclusion** In the meantime, the guidance from the British Society of Periodontology and Implant Dentistry is to ensure patients understand the lack of information and research available. The National Institute for Health and Care Excellence support this and note that there may be a place for e-cigarettes as an aid to quitting smoking, supporting patients to become tobacco-free. It would, however, be sensible for dental practitioners to discourage negative health habits, including e-cigarette use, unless to replace a potentially more dangerous habit, such as conventional smoking.

## Key points

- Summarises the current evidence on the effect of e-ciagrette usage on periodontal health and limitations in this area of study.
- Educates on the possible effects of e-cigarette usage on periodontal tissues, periodontal health and periodontal treatment outcomes, and compares to conventional smoking and non-smoking.
- Addresses the discrepancy between public perception of e-cigarette usage and the findings of current evidence.
- Provides a summary of current guidelines and advice to practitioners.

## Introduction

The prevalence of e-cigarette use (vaping) has increased significantly, with record rates of use reaching 9.1% in the United Kingdom (UK).^1^ Given that e-cigarettes were introduced in the early 2000s, scientific literature on this subject is limited compared to that of conventional smoking. However, there are emerging trends in the literature, explored in this narrative review, which inform the current guidance that clinicians should implement in practice.

## Aim

To educate clinicians on the current literature regarding the impacts of e-cigarette usage on periodontal health and its limitations, including current guidance provided.

## Methods

This narrative review was developed as a follow-on from the Student British Society of Periodontology and Implant Dentistry (BSP) webinar, ‘E-cigarette vaping: a risk for periodontal health? By students, for students', which was presented by the authors of this paper. A literature search was conducted using PubMed and Google Scholar. Search terms included combinations of ‘e-cigarette', ‘vaping', ‘systemic effects', ‘periodontal health', ‘periodontal status', ‘periodontal treatment', ‘implant outcomes', and ‘smoking cessation'. Given the limited number of studies and systematic reviews available in this area, a narrative review approach was considered appropriate to synthesise the existing literature. To inform the ‘Practitioner guidance' section, the latest guidelines and recommendations were sourced from the official websites of the National Institute for Health and Care Excellence (NICE), the BSP, and the *Delivering Better Oral Health* (*DBOH*) toolkit published by the Office for Health Improvement and Disparities, Department of Health and Social Care, NHS England and NHS Improvement (these are correct as of the date of manuscript submission).

## Characteristics of e-cigarette users

Most e-cigarette users are current or previous smokers; usage among never-smokers has been reported to be rare.^2^ Use among men is more common, with 9.5% of men 16 years and older reporting e-cigarette use daily or occasionally, compared to 7.9% of women.^3^ E-cigarette use is fairly evenly distributed between ages 16–59, with a reduced rate of use in those over 60.^3^ Younger individuals tend to use e-cigarettes for enjoyment whereas individuals older than 45 years are more likely to use e-cigarettes to quit smoking.^4^ Box 1 highlights additional demographic trends and usage patterns among e-cigarette users.^4^^,^^5^

It is important to consider that not all e-cigarettes are created equal. They vary in nicotine levels and flavours – each with different ingredients – offered by companies to appeal to a plethora of users, leading to diverse effects across the population.

Box 1 Additional facts about e-cigarette users

- 40.6% of older people prefer tobacco-flavoured e-cigarettes versus only 4.8% of younger people; 51.1% of younger people prefer fruit flavours^4^
- Being young and perceiving e-cigarette use as less harmful than conventional smoking increases the chance of recent (within the last 30 days) e-cigarette use^4^
- Women from white and multiracial backgrounds were more likely to have tried e-cigarettes^5^
- Lowest use was among Black African and Indian men, and women from all Asian and Black African backgrounds^5^
- E-cigarette users are more likely to have parents with a lower socioeconomic status at birth^2^

## E-cigarettes and systemic links

**Fig. 1:**
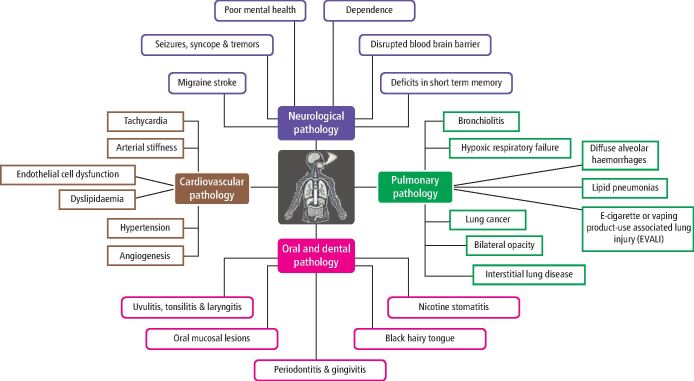
Confirmed or potential oral and systemic associations of e-cigarette use suggested in various papers. Please note varying degrees of support for these conditions – not conclusive evidence^6^^,^^7^^,^^8^

Evidence on the systemic effects of e-cigarette use does not have a robust evidence base – there is a distinct lack of long-term studies on this subject. There is, however, emerging evidence that e-cigarette use has neurological, pulmonary, cardiovascular and oral and dental effects, as detailed in Figure 1.^6^^,^^7^^,^^8^ Daily or heavy e-cigarette users also tend to have higher rates of alcohol and cannabis use and other high-risk behaviours,^9^ which can influence health outcomes and are considered co-morbidities for many diseases. The lack of long-term human studies on e-cigarette use must be addressed to better inform users, public health policies, and resource allocation.

## Potential mechanisms of periodontal deterioration of e-cigarette users

In a systematic review, Charde *et al.*^10^ highlighted several mechanisms by which e-cigarette use may contribute to periodontal disease. They noted elevated inflammatory biomarkers (IL-1β, IL-6, TNF-α, IL-4, and IL-8) in gingival crevicular fluid and saliva, indicating an inflammatory response akin to traditional smoking. This persistent inflammation can lead to accelerated bone resorption and tissue degradation – key features of periodontal disease. Additionally, the review found increased levels of periodontal pathogens, particularly *Porphyromonas gingivalis* and *Fusobacterium nucleatum*, which are linked to periodontitis. These results align with findings from Ganesan *et al.*,^11^ who demonstrated that e-cigarette aerosol exposure increases these pathogenic bacteria. This microbial imbalance may heighten the risk of periodontal infections and tissue destruction, reinforcing the link between e-cigarette use and periodontal disease progression.

## Comparison of e-cigarette use, conventional smoking and non-use/non-smoking

### Prevalence of periodontitis

Studies on the association between e-cigarette use and periodontal disease show mixed results. From the current evidence base, various systematic reviews and cross-sectional studies have suggested that e-cigarette use is less harmful to periodontal health than smoking conventional cigarettes, with e-cigarette users generally having lower plaque index scores, probing pocket depths (PPDs), attachment loss, marginal bone loss and lower levels of pro-inflammatory mediators than smokers.^12^^,^^13^^,^^14^ However, it should be emphasised that most studies included in these systematic reviews are observational in nature. While such studies can demonstrate associations, they are limited in establishing a causative relationship between e-cigarette use and periodontal health. Additionally, the lack of long-term studies means that the chronic effects of e-cigarette use on periodontal health remain unclear.

Despite the current evidence indicating that e-cigarette use presents a lower risk to periodontal health than smoking, e-cigarette use has still been linked to increased prevalence of periodontitis compared to non-smokers/non-users, as demonstrated by Jeong *et al.*^15^ Notably, e-cigarette users comprised only 1.6% of the sample (222 participants), while the remaining 98.4% were either smokers or non-smokers, which may affect the statistical power of these results. Additionally, concurrent use of other tobacco products was not noted, and this study relied on self-reported data for oral health status, which could have introduced recall or social desirability bias.

Therefore, it is reasonable to suggest from current evidence that e-cigarette use could potentially be less harmful than conventional smoking to the periodontium but may still present a risk to periodontal health. More studies with higher statistical power and more rigorous methods will be needed to improve the strength of these findings.

### Severity of periodontitis

Clinical attachment loss (CAL) is a critical parameter for assessing the severity of periodontal disease, reflecting both historical and current extent of tissue destruction. While individual studies, such as Xu *et al.*,^16^ have reported mean CAL values of 2.9 mm for e-cigarette users, 2.2 mm for non-smokers, and 3.5 mm for smokers, these findings should be interpreted with caution due to potential confounding factors, including lack of comment on prior smoking history and limitations in cross-sectional design of the study. Recent systematic reviews and meta-analyses have collated evidence. For instance, Shabil *et al.*^17^ analysed 12 studies and found a non-significant increase in CAL among e-cigarette users compared to non-smokers, with a pooled mean difference of 0.120 mm (95% CI -0.045–0.285; I² = 90%). A separate, very recent meta-analysis reported that e-cigarette users had a greater risk of periodontal disease than non-smokers but a lower risk than cigarette smokers.^18^ These findings suggest that while e-cigarette users may experience greater CAL than non-smokers, the evidence is not conclusive, and further longitudinal studies are necessary to establish a causal relationship between e-cigarette use and periodontal health deterioration.

### Response to non-surgical periodontal treatment

A retrospective exploratory study by Shah *et al.*^19^ compared four groups following non-surgical periodontal treatment (NSPT): former smokers, former smokers who currently use e-cigarettes, current smokers, and non-smokers. Former smokers who were now e-cigarette users showed a less favourable response to NSPT compared to both former smokers and non-smokers, including deeper PPD and a greater need for surgical intervention. Interestingly, the clinical outcomes for this group were similar to that of the current smoker group, therefore suggesting that e-cigarette use in former smokers may compromise periodontal healing to a similar extent as continued conventional cigarette smoking.

It is important to note that Shah *et al.*'s study was exploratory and not statistically powered. Additionally, the confounding effects of previous tobacco use remain a limitation that was not controlled in this study. Therefore, the findings of this study should be seen as suggestive rather than definitive. More research is needed to assess periodontal treatment outcomes not only in e-cigarette users with a history of smoking, but also in e-cigarette users who have never smoked conventional cigarettes.

### Implant outcomes

Two small observational studies, by ArRejaie *et al.*^20^ and AlQahtani *et al.*,^21^ compared peri-implant health among exclusive cigarette smokers, exclusive e-cigarette users, and non-smokers with implants in function for at least three years. Both studies found that e-cigarette users exhibited worse clinical and inflammatory peri-implant profiles than non-smokers, though generally less severe than those seen in cigarette smokers. E-cigarette users showed increased PPD, greater plaque accumulation, more marginal bone loss, and higher levels of pro-inflammatory cytokines (IL-1β, MMP-9, TNF-α) than non-smokers, but lower levels than cigarette smokers.

In the study by ArRejaie *et al.*, the e-cigarette group consisted of exclusive e-cigarette users, with dual users explicitly excluded. However, the criteria used to define the e-cigarette group in AlQahtani *et al.*'s study were less clearly described, making it possible that dual users were included, which could be a major confounder. Additionally, neither study accounted for participants' former smoking history, which is another confounding factor. Both studies also relied on self-reported smoking behaviour without biochemical verification, which could introduce bias, and only studied male participants, which limit the generalisability of the results. Furthermore, the studies did not assess long-term implant survival or the progression of peri-implant disease. However, their findings do highlight the need for further investigation through well-designed, controlled studies to provide further evidence on the impact of e-cigarette use on implant outcomes.

### Response to peri-implantitis treatment

Research in this area remains extremely limited, and existing studies have notable methodological weaknesses.

Nevertheless, a small, observational case-control study^22^ has suggested that peri-implantitis treatment is less effective in e-cigarette users compared to conventional smokers and non-smokers. In this study, three groups of patients – exclusive conventional cigarette smokers, exclusive e-cigarette users, and non-smokers – each with one infected implant, underwent surgical therapy (open flap debridement with implant surface modification and bone recontouring) following initial non-surgical treatment. Post-treatment, clinical parameters (gingival colour and consistency, plaque index, bleeding on probing [BoP], PPD, keratinised tissue width) and salivary inflammatory biomarkers (MMP-8, IL-6, IL-1β, TIMP-1) were assessed at three time points: one month, six months, and one year. E-cigarette users in this study generally exhibited higher clinical and salivary inflammatory markers than never-smokers and cigarette smokers during the follow-up period.

However, the sample size of this study was very small, with only 60 participants, with 20 in each study group. There is also a lack of detail on power calculations or effect size assumptions, limiting the significance of these findings. Confounding variables were not controlled for, and habits were self-reported, raising the potential for recall and social desirability bias. Given these limitations, further well-powered studies are necessary before definitive conclusions can be drawn on this subject.

## What happens to periodontal health when a smoker switches to e-cigarette use?

Most e-cigarette users in the UK are either current or former smokers,^3^ making the impact of switching from smoking to e-cigarette use a particularly important area of study.

There is limited evidence on the changes that occur in the periodontium when a conventional smoker switches to e-cigarette use. Preliminary evidence suggests that switching may improve periodontal health, as found in a systematic review by Yang *et al.*;^13^ although, it was noted in the review that the evidence included in the review was weak to moderate. Tatullo *et al.* observed an improvement in clinical periodontal parameters (BoP, papillary bleeding index and plaque levels) among individuals who switched from conventional cigarettes to e-cigarettes. The results were measured over a four-month period.^23^ Potential confounding factors, such as the Hawthorne effect and conventional smoking relapse, were accounted for; participants were required to report their e-cigarette and smoking habits in a personal diary and asked not to alter their oral hygiene habits; and Smoke Check tests were implemented to check that participants had not smoked a conventional cigarette within the last 24–48 hours. These measures improve the reliability of this study.

A pilot study by Wadia *et al.*^24^ found that BoP (a clinical marker of periodontal disease) increased after participants switched from smoking to e-cigarettes. This finding, however, may not suggest that switching from smoking to e-cigarette use is worse for periodontal health. BoP levels in Wadia *et al.*'s data doubled between baseline and the two-week follow-up, similar to patterns seen in individuals who quit smoking without using e-cigarettes.^25^^,^^26^ This suggests that switching to e-cigarettes may not alter the typical physiological response to smoking cessation in the periodontium.

Both Tatullo *et al.* (n = 110) and Wadia *et al.* (n = 20) conducted small-scale studies. Tatullo *et al.* focused solely on clinical indices and Wadia *et al.* primarily assessed BoP as the main outcome. As such, there is a need for large-scale, high-quality studies that measure other clinical and biological markers of periodontal inflammation and disease, especially given that e-cigarettes are now recognised as an effective smoking cessation aid^27^ and may soon be more widely distributed through NHS (National Health Service) initiatives such as the Swap to Stop Programme.^28^

## Practitioner guidance

### *Delivering Better Oral Health* guidance

Guidance on e-cigarette usage and potential effects on the periodontium are covered in limited detail in Chapter 11^29^ and Chapter 15^30^ of *DBOH*, respectively. The guide emphasises the role of e-cigarettes as a stop-smoking aid but highlights that e-cigarettes are not classified as licensed medicines. It also advises that e-cigarette users are at a greater risk for periodontal disease compared to non-smokers, but at lower risk compared to tobacco users. Therefore, e-cigarettes can be used as a transition tool in smoking cessation.

**Fig. 2:**
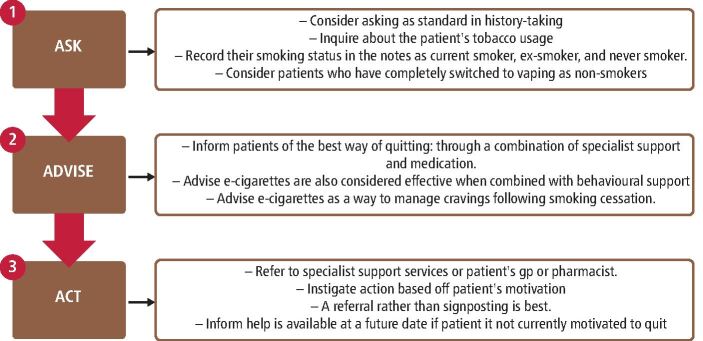
Flowchart of the ‘Very Brief Advice' framework with an accompanying explanation of each stage^29^

A quick toolkit provided in *DBOH* to increase chances of successful smoking cessation for all patients is Very Brief Advice (Fig. 2). Dental practitioners should Ask, Advise, and Act at every opportunity or recall appointment for all adults and adolescents.

### British Society of Periodontology and Implant Dentistry guidance

The BSP provides limited guidance for practitioners regarding e-cigarettes in their *Good Practitioners Guide* (2016).^31^ They suggest that e-cigarettes are likely to be less harmful to periodontal tissues compared to conventional cigarettes. Practitioners should inform patients of the current knowledge gap and that the full effect of e-cigarettes is still to be investigated. The recent BSP article, ‘Is vaping harmful to oral health?',^32^ discourages e-cigarette use among non-smokers, particularly adolescents, but endorses their use as a cessation tool for smokers, recommending that dental professionals support patients who choose this option.

### National Institute for Health and Care Excellence guidance

The *Tobacco: Preventing Uptake, Promoting Quitting and Treating Dependence* guide published by NICE states that e-cigarettes are not licensed by the Medicines and Healthcare products Regulatory Agency as a stop-smoking medicine.^33^

The guidance for those who provide stop-smoking support and advice is as follows:

- Advise there is not enough evidence to inform of the long-term harm of e-cigarettes
- Advise e-cigarettes are likely to be substantially less harmful than smoking
- Advise that use of e-cigarettes should be accompanied by complete cessation of tobacco smoking
- Discuss the duration the patient plans to use nicotine containing e-cigarettes
- Discuss using e-cigarettes long enough to prevent a return to smoking
- Discuss how to stop e-cigarette use when the patient is ready to.

## Public perception

As highlighted in this review, current evidence suggests that e-cigarettes are a safer alternative to traditional smoking. However, public perception of e-cigarette use does not always align with scientific evidence. According to a 2023 survey by Action on Smoking and Health, 43% of adults believe that e-cigarettes are as harmful, if not more harmful, than conventional cigarettes.^1^

Over the past decade, public perception has grown more negative, with a larger proportion of the population now viewing e-cigarette use as equally or more dangerous than smoking. This disconnect between public opinion and research findings may be partially attributed to selective media coverage. Research that highlights potential dangers of e-cigarettes tends to receive greater media attention, while studies demonstrating minimal or no harm are often under-reported. The focus on negative outcomes can skew public understanding and amplify fears surrounding e-cigarette use, further contributing to the misperception that e-cigarettes are just as harmful as traditional cigarettes.

As long-term data are still emerging, there remains uncertainty regarding the long-term effects of e-cigarette use. This ambiguity may reinforce the cautious, often negative view held by many individuals.

For these reasons, it is essential to provide balanced and accurate public health information, clearly communicating both the risks associated with e-cigarette use and how they compare to those of smoking. This will allow individuals to make more informed decisions about their health, particularly regarding the impact on oral and periodontal health.

## Research limitations in e-cigarette and periodontal health studies

A key limitation in current research is the lack of long-term data, as most studies rely on short-term observational designs. Randomised controlled trials (RCTs) are needed to provide clearer evidence on the specific impacts of e-cigarette use, separate from confounding variables.

One critical issue is the lack of differentiation between ‘naive e-cigarette users' – those who have never smoked – and former or current smokers. Many studies fail to account for prior smoking history, which is a major confounding factor. Former smokers may already have pre-existing periodontal damage, complicating the interpretation of results. The inclusion of such individuals in study cohorts can obscure the specific effects of e-cigarettes on periodontal health, leading to potentially biased conclusions.

Additionally, the rapid evolution of e-cigarette products poses challenges for research, making it difficult to generalise findings across all types of e-cigarettes. Newer devices with different nicotine delivery systems, aerosol compositions, and usage patterns necessitate more rigorous methodologies in future research, including longitudinal studies and RCTs, to better understand the long-term risks of e-cigarette use on periodontal health.

## Conclusion

Clinicians should be aware of the possible detrimental effects of e-cigarette use on the periodontium, while acknowledging that current evidence suggests that e-cigarette use may be less harmful than smoking. Given the prevalence of e-cigarette use among smokers and its role in NHS smoking cessation efforts, it is essential to provide accurate advice, ensuring patients are not dissuaded from a potentially safer harm reduction option. Current practice guidelines are aptly summarised in this quote by England's ex-Chief Medical Officer, Chris Whitty: ‘if you smoke, vaping is much safer. If you don't smoke, don't vape'.^34^

## Data Availability

No new data were generated or analysed during the writing of this review. Therefore, data sharing is not applicable for this article.
